# Common Misconceptions about Diet and Breast Cancer: An Unclear Issue to Dispel

**DOI:** 10.3390/cancers16020306

**Published:** 2024-01-11

**Authors:** Anastasia Lalioti, Laura Verzeletti, Paola Tiberio, Riccardo Gerosa, Mariangela Gaudio, Giuseppe Saltalamacchia, Manuela Pastore, Alberto Zambelli, Armando Santoro, Rita De Sanctis

**Affiliations:** 1 Department of Biomedical Sciences, Humanitas University, Via Rita Levi Montalcini 4, 20072 Pieve Emanuele, Italy; anastasia.lalioti99@gmail.com (A.L.); laura.verzeletti@st.hunimed.eu (L.V.); riccardo.gerosa@cancercenter.humanitas.it (R.G.); mariangela.gaudio@cancercenter.humanitas.it (M.G.); alberto.zambelli@hunimed.eu (A.Z.); armando.santoro@hunimed.eu (A.S.); rita.de_sanctis@hunimed.eu (R.D.S.); 2 Medical Oncology and Hematology Unit, IRCCS Humanitas Research Hospital, Via Manzoni 56, 20089 Rozzano, Italy; giuseppe.saltalamacchia@cancercenter.humanitas.it (G.S.); manuela.pastore@humanitas.it (M.P.)

**Keywords:** nutrition, sugar, soy, dairy product, breast cancer risk, menopause, molecular subtypes, hyperinsulinemia, metabolism, obesity

## Abstract

**Simple Summary:**

Breast cancer is the most prevalent cancer among women. Extensive research has been conducted on eating patterns, and increasing evidence suggests that diet significantly influences the development, progression, and prevention of breast cancer. However, besides the well-known role of alcohol and red and processed meat, the impact of other dietary components remains a subject of debate. Specifically, the potential connection between sugar, dairy components, and soy with breast cancer risk raises questions. Focusing on the studies conducted in the last decade, our literature review shows a negative association between breast cancer incidence and both dairy product and soy consumption, while complex data emerged about sugar intake by itself. However, high heterogeneity across studies’ findings was observed, thus highlighting the need for comprehensive investigations considering both patient- and cancer-related factors to develop preventive strategies that should be incorporated into international guidelines.

**Abstract:**

Breast cancer (BC) constitutes a prevalent health condition among women. Recent years have witnessed the identification of dietary proto-oncogenic factors that deserve attention. Besides the well-known role of alcohol and red and processed meat in BC development, the impact of other dietary components remains unclear. Our narrative review aims to explore the diet-BC relationship, focusing on sugar, dairy, and soy consumption. We conducted a PubMed literature search covering the last decade (2013–2023) and included 35 papers. We found limited evidence on the association between high sugar intake and BC incidence. On the other hand, dairy and soy consumption displayed a protective effect in the majority of the analyzed papers. However, a significant degree of heterogeneity was reported among the results. Menopausal status and the specific BC molecular subtypes were the main factors influencing the interpretation of the results. Exploring dietary factors and BC revealed inconsistencies: high glycemic index post-menopause may be a risk factor, while sugar-sweetened drinks and artificial sweeteners yielded conflicting results; fermented dairy showed potential benefits, non-fermented dairy presented inconsistent findings; soy impact on BC varied according to molecular subtype, with some studies suggesting a positive association in luminal-like BC. Hence, further investigation is crucial to obtain a uniform consensus on the diet-BC relationship.

## 1. Introduction

Breast cancer represents a significant global health concern, being the most frequent tumor in women worldwide. In fact, it affects millions of women, with 287,850 new cases estimated in 2022 in the United States [1]. In women, breast cancer incidence has been slowly increasing throughout the last years (approximately 0.5% per year from the mid-2000s), currently accounting for almost one-third of all new cancer diagnoses (followed by lung and colorectal cancers). This could be partially ascribed to the decline in the fertility rate and the overall increase in body weight. Nevertheless, thanks to advances in early detection, surgical techniques, and targeted therapies, mortality rates have continuously decreased throughout the last decades. Presently, the 5-year relative survival rate for breast cancer, encompassing all stages, stands at approximately 90%. However, an estimated 43,250 female deaths occur due to breast cancer in the United States every year [1].

In the quest to understand breast cancer causes, epidemiologic studies have unveiled a multitude of risk factors, which are defined as modifiable and non-modifiable. These factors include genetic predisposition, early menarche, delayed menopause, advanced age at first childbirth, reduced childbirths, limited breastfeeding, menopausal hormone replacement therapy, and lifestyle elements, such as alcohol consumption, excess body weight, and physical inactivity [2,3]. Notably, the American Society of Clinical Oncology (ASCO) guidelines emphasize lifestyle choices like obesity, excessive alcohol consumption, smoking, and sedentary living, alongside environmental exposures (e.g., pesticides or radiation) and dietary habits as relevant modifiable risk factors. Further bolstering breast cancer prevention, strategies advocate for regular physical exercise and maintaining a healthy weight [4,5].

Recent years have witnessed the identification of dietary proto-oncogenic factors that deserve attention [6,7,8]. Specifically, excessive alcohol consumption consistently emerges as a significant contributor to breast cancer risk [7,9]. Diets rich in saturated and trans fats, often found in red meat and processed foods, have also come under scrutiny for their potential role in elevating risk [8]. Remarkably, the World Health Organization’s International Agency for Research on Cancer designates red and processed meats as probable and established carcinogens, respectively [9]. Conversely, embracing a diet rich in fruits and vegetables, brimming with antioxidants and protective compounds, has been associated with a reduced risk [10,11,12,13]. While these factors alone do not cause breast cancer, reducing alcohol and red/processed meat consumption and increasing fruit and vegetable intake can be valuable steps toward lowering risk and promoting overall well-being [14,15].

However, the relationship between cancer development and other dietary components remains unclear. The biological basis may depend on elevated estrogen levels, pro-inflammatory molecules, and the impairment of glucose metabolism. The association between breast cancer and estrogen levels is especially direct for luminal-like tumors. Factors implicated in obesity, such as chronic low-grade inflammation and hyperinsulinemia, may also contribute to carcinogenesis [16]. Not only can excessive sugar consumption lead to weight gain, obesity, and metabolic syndromes, which increase the oncological risk [17,18,19], but it can also cause a disruption in hormone levels and promote insulin resistance, contributing to breast cancer development [20]. Therefore, limiting sugar intake could be a prudent strategy for breast cancer prevention [21]. Similarly, the role of dairy products in breast cancer remains contentious since only a few studies have highlighted a positive correlation, particularly among post-menopausal women [22]. Lastly, the intriguing idea that soy, rich in phytoestrogens, may influence hormone-sensitive breast tumors has sparked extensive research, yielding inconsistent results. While some studies have found no significant association between soy intake and breast cancer incidence or recurrence, others have suggested a protective effect, particularly in populations with traditionally high soy consumption, such as in Asia [23,24].

Despite a wealth of research investigating the interplay of diet and breast cancer, consensus on the role of sugar, dairy products, and soy in breast cancer incidence remains elusive. Therefore, we hypothesized that by updating the available information on these fields, we could be able to dispel these intricate associations more effectively. Thus, the objective of this narrative review is to summarize the recent findings from selected studies published in the last decade that explore the diet-BC relationship, with a focus on sugar, dairy, and soy consumption.

## 2. Methods

To perform the present narrative review, a literature search of the PubMed database was conducted with the following search terms: “breast cancer” OR “breast carcinoma” OR “breast neoplasm” AND “risk” OR “incidence” OR “development” AND “sugar” OR “sweeteners” OR “dairy” OR “milk” OR “soy” OR “isoflavone”. The research covered the last ten years (2013–2023). As publication types, we considered “article” OR “meta-analysis”, and we selected English as the language. Duplicate and irrelevant papers were omitted (e.g., studies describing trials in progress, papers analyzing biomarker levels in response to food intake, and research articles addressing associations different from breast cancer incidence). The selected papers only focused on the risk of breast cancer development in association with sugar, dairy, or soy consumption. Studies regarding correlation with the response to treatment and/or prognosis were excluded. Overall, a total of 35 papers were reviewed in the present narrative review, allowing us to provide a comprehensive and up-to-date overview of the current knowledge on this topic and valuable insights into the complexities of diet-cancer interactions.

## 3. Sugar Intake

Recent years have witnessed a surge in interest surrounding the intriguing connection between sugar intake and the risk of breast cancer (Table 1), driven by the well-established association between weight gain and the incidence of breast cancer [17,18].

Outcomes vary depending on the menopausal status of participants. A comprehensive meta-analysis involving 14 prospective cohort studies [25], encompassing a total of 15,839 cases and 577,538 participants, delved into the relationship between glycemic index (GI) and glycemic load (GL) and breast cancer development. Surprisingly, it unveiled a dose-dependent protective effect when the GI was above 67 units/day (relative risk [RR] 1.05, 95% confidence interval [CI] 1.01–1.09, *p* = 0.008). However, in a subgroup analysis evaluating menopausal status, a positive association between breast cancer development and GI was only observed in the post-menopausal setting (RR 1.06, 95% CI 1.00–1.13, *p* = 0.044) and not in the pre-menopausal one (RR 1.06, 95% CI 0.95–1.17, *p* = 0.282).

In contrast, a meta-analysis by Mullie P. et al. [26], spanning 12 cohort studies conducted from 2003 to 2011 and including a total of 773,971 women, showed a weak association between high GI and GL and breast cancer risk (RR 1.05, 95% CI 1.00–1.11 and RR 1.06, 95% CI 1.00–1.13, respectively). The results of their subgroup analyses indicated that there was no significant impact of menopausal status on this association. Pre-menopausal women (from five included studies) and post-menopausal women (from nine included studies) had comparable RRs for elevated GI and GL (RR 1.04, 95% CI 0.86–1.27, and 1.05, 95% CI 0.98–1.13, respectively) and 1.23, 95% CI 0.75–2.00 and 1.05, 95% CI 0.97–1.13, respectively).

Other studies reported the potential association between sugar intake and specific breast cancer subtypes. A systematic review and dose-response meta-analysis [27], including a total of 892,403 women, showed a statistically significant positive association between estrogen receptor (ER)-negative subtype and 50 units/day of GL in post-menopausal women (RR 1.28, 95% CI 1.08–1.52, *p* = 0.05).

Shifting the focus to sugar intake from beverages, in a prospective study cohort examining 35,593 participants, the authors highlighted that the consumption of one to six sugar-sweetened soft drinks per week was positively associated with obesity-related cancers, including post-menopausal breast cancer (Hazard Ratio [HR] 1.21, 95% CI 1.03–1.43) [28]. Notably, artificially sweetened drinks failed to show a statistically significant link. Moreover, in a systematic review and dose-response meta-analysis by Li Y. et al. [21], sugar-sweetened beverage (SSB) consumption exhibited a positive association with overall cancer risk (highest versus lowest category RR 1.12, 95% CI 1.06–1.19, *p* < 0.001), with subgroup analyses indicating this association in breast cancer patients (number of studies included: 7, RR 1.21, 95% CI 1.02–1.43, *p* = 0.027). In contrast, daily fruit juice intake displayed no statistically significant association with breast cancer risk (number of studies included: 3, RR 1.06, 95% CI 0.93–1.20, *p* = 0.375). However, the inclusion of only three cohort studies constituted the primary limitation of the meta-analysis. A prospective French cohort study [29] involving 783 cases highlighted that added sugar consumption was linked to a higher risk of breast cancer (HR for fourth quartile vs. first quartile 1.47, 95% CI 1.12–1.91, *p* = 0.02). This correlation was more pronounced in pre-menopausal patients (HR for fourth quartile vs. first quartile 1.95, 95% CI 1.24–3.06, *p* = 0.002 and 1.41, 95% CI 0.93–2.14, *p* = 0.05, for total and added sugar, respectively). In contrast, the trend observed in post-menopausal breast cancer patients was comparable but did not reach statistical significance.

Adding to the complexity, the sources of sugar were also scrutinized in the NutriNet-Santé cohort, which enrolled 79,742 patients between 2009 and 2019. A statistically significant increase in breast cancer prevalence was observed in a dose-response fashion with sugary drinks (*p* = 0.002), dairy products (*p* = 0.01), milk-based desserts (*p* = 0.02), non-fruit dietary sources (*p* = 0.0007), solid foods (excluding sugary drinks; *p* = 0.003), and free sugars (*p* = 0.01) [29]. In a subsequent investigation of the NutriNet-Santé population, the authors observed that artificial sweeteners, particularly aspartame and acesulfame K, were positively associated with an elevated risk of breast cancer (*n* = 979 cases, HR 1.22, 95% CI 1.01–1.48, *p* = 0.036, for aspartame and HR 1.13, 95% CI 1.01–1.26, *p* = 0.007 for acesulfame K) [30].

In summary, the current body of evidence is still limited and equivocal regarding the possible association between high sugar intake and the increased risk of developing breast cancer. Well-designed prospective studies, clinical trials, and population-based research are imperative to unravel this intricate puzzle. Future studies should consider various types of sugars, their sources (natural vs. added sugars), dietary patterns, and other lifestyle factors. Furthermore, stratifying patients according to receptor status and menopausal status might help to gain a more comprehensive understanding of the complex interplay between sugar and breast cancer.

**Table 1:** Main findings on the association between sugar intake and breast cancer development.

Author (Year) [Ref]	Food/Intervention under Investigation	Type of Study	Participants	Assessment of Dietary Intake	Main Results
Mullie P et al. (2016) [26]	GI and GL	Meta-analysis	773,971 women	FFQ	Women with a high GI or GL have a 5–6% increased risk of breast cancer
Schlesinger S et al. (2017) [27]	Carbohydrate GI, GL diet	Systematic review and dose-response meta-analysis	892,403 women	FFQ	GL and carbohydrate intake were associated with an increased risk of breast cancer only among hormone receptor–negative tumors, particularly ER-negative.
Hodge AM et al. (2018) [28]	Artificially sweetened soft drinks	Prospective cohort study	35,593 participants	FFQ	The highest risk of breast cancer was associated with 1–6 sweetened soft drinks/week in post-menopausal women.
Debras C et al. (2020) [29]	Added sugar intake	Prospective cohort study	101,279 participants	Repeated 24-h dietary records	Total sugar intake was positively associated with high overall cancer risk, including breast cancer.
Li Y et al. (2021) [21]	Sugar-sweetened beverages and fruit juice	Systematic review and dose-response meta-analysis	8465 cases and 119,153 controls	FFQ	The highest level of sugar-sweetened beverage consumption showed an increased breast cancer risk.
Long T et al. (2022) [25]	GI and GL	Meta-analysis	15,839 cases and 577,538 participants	FFQ	A positive association between breast cancer development and GI was observed only in the post-menopausal setting.
Debras C et al. (2022) [30]	Artificial sweeteners	Prospective cohort study	102,865 adults	Repeated 24-h dietary records	Artificial sweeteners (aspartame and acesulfameK) were associated with increased breast cancer incidence.

GI: glycemic index; GL: glycemic load; FFQ: food frequency questionnaire.

## 4. Dairy Consumption

Over the past decade, investigations into the relationship between dairy product consumption and the risk of breast cancer have yielded disparate outcomes, introducing complexity into this scientific inquiry (Table 2).

Crucially, the outcomes exhibited variability contingent upon the menopausal status of participants. The cohort study led by Shin et al. [31] involving 78,320 Korean women found that in pre-menopausal women (<50 years old, *n* = 29,803), a high daily intake of milk (≥1 serving/day) was inversely associated with breast cancer development (HR 0.58, 95% CI 0.35–0.97, *p* = 0.0195), compared to women of the same age group consuming <1 serving/week (21 versus 66 breast cancer cases, respectively). However, this protective effect was not confirmed for women older than 50 years of age (53 versus 93 breast cancer cases, *p* = 0.6). Zang and colleagues, in a systematic review and meta-analysis across twenty-two prospective cohort studies (1,566,940 participants) and five case-control studies (33,372 participants), highlighted this inverse association, discerning a protective role solely in pre-menopausal women (RR 0.88, 95% CI 0.77–1.00, *p* = 0.057) [32]. Differently, the cohort study by Couto et al. [33], including 44,840 women, reported a statistically significant inverse association between dairy consumption and breast cancer risk in both pre-menopausal and post-menopausal women adhering to a Mediterranean diet (for 290 g/day of dairy intake, RR 0.93, 95% CI 0.87–0.99, RR 0.93, 95% CI 0.86–0.99, and RR 0.89, 95% CI 0.87–0.99 in all women, pre-menopausal and post-menopausal, respectively). On the contrary, Fraser et al. [34], in a prospective cohort study with 52,795 North American women, observed no discernible difference in breast cancer risk between pre-menopausal and post-menopausal cohorts. Nevertheless, the authors noted a HR of 1.23 and 1.54, respectively, for the 10th and the 90th percentiles of dairy consumption, emphasizing the influential role of the intake amount in breast cancer onset. The prospective study cohort by Marcondes et al. [35] did not ascertain a correlation between breast cancer risk and dairy products in post-menopausal women (HR 1.60, 95% CI 1.07–2.39, *p* = 0.50). Examining dairy consumption in adolescence and early adulthood, Farvid et al. [36] examined 90,503 pre-menopausal women and did not establish a statistically significant link in the pre-menopausal setting (total dairy HR 1.02, 95% CI 0.97–1.07; for early adulthood total dairy HR 1.01, 95% CI 0.97–1.04).

Regarding breast cancer subtypes, in the above-mentioned cohort study by Farvid and colleagues [36], a positive correlation emerged between dairy intake and hormone receptor-negative breast cancer (each serving/day: total dairy HR 1.11, 95% CI 1.00–1.24; high-fat dairy HR 1.17, 95% CI 1.04–1.31), countered by a negative correlation with ER and progesterone receptor (PgR)-positive subtypes (each serving/day HR 0.91, 95% CI 0.86–0.97). In contrast, Fraser et al. [34], by analyzing 52,795 North American women, observed an escalated risk of hormone receptor-positive subtype development with higher dairy intake (HR 1.29, 95% CI 1.07–1.56, *p* = 0.007 and 1.43, 95% CI 1.11–1.83, *p* = 0.007). On the other hand, in the prospective cohort study by Genkinger et al. involving 52,062 African American women [37], the authors detected a negative association only between >250 g/week intake of whole milk compared to 0 g/week and hormone receptor-negative subtypes (ER-negative: RR 0.33, 95% CI 0.13–0.84, *p* < 0.05, and PgR-negative: RR 0.49, 95% CI 0.24–0.99, *p* = 0.11).

A granular exploration of the distinct components of dairy products, including milk fermentation, fat content, and dairy product types, revealed intriguing patterns. Focusing on milk fermentation, the meta-analysis by Yujing He et al. [22], including 27 studies, showcased the non-statistically significant protection of non-fermented dairy products (HR 0.99, 95% CI 0.94–1.03, *p* = 0.54), in contrast to the statistically significant protective effect of fermented dairy products observed only in post-menopausal women (6 studies included, HR 0.96, 95% CI 0.93–0.99, *p* = 0.021). Delving into these complex aspects, an analysis of 11 studies investigating the impact of low-fat dairy products on breast cancer risk revealed a statistically significant protective effect of low-fat products solely in pre-menopausal women (HR 0.94, 95% CI 0.89–1.00, *p* = 0.048). Similarly, a case-control study involving 275 Iranian women [38] showed an 85% reduction in breast cancer risk associated with high total dairy intake (odds ratio [OR] 0.14, 95% CI 0.04–0.38, *p* < 0.001), especially for fermented dairy products (OR 0.26, 95% CI 0.09–0.72 and OR 0.06, 95% CI 0.02–0.19 in the third and fourth quartiles, respectively; *p* = 0.001 for both), as well as for low-fat dairy products (OR 0.25, 95% CI 0.08–0.81 and OR 0.10, 95% CI 0.03–0.34 in the third and fourth quartiles, respectively; *p* = 0.003 for both). Partially in line with these findings, the prospective cohort study of Aguilera-Buenosvinos et al. [39], involving 10,930 women belonging to the ‘Seguimiento Universidad de Navarra’ project, revealed a risk reduction in pre-menopausal women consuming 1–2 servings of low-fat dairy products per day (adjusted HR Q2 vs. Q1 0.26, 95% CI 0–0.59, *p* = 0.001; adjusted HR Q3 vs. Q1 0.48, 95% CI 0.25–0.92, *p* = 0.027), whereas 2–4 servings of dairy products yielded a risk reduction in the post-menopausal setting (adjusted HR Q2 vs. Q1 0.28, 95% CI 0.10–0.76, *p* = 0.012; adjusted HR Q3 vs. Q1 0.42, 95% CI 0.18–0.96, *p* = 0.040).

Finally, considering dairy product types, inconsistent results were obtained when yogurt, milk, and cheese were specifically analyzed [31,32,34,36,40], although the majority of papers suggested that their intake was not associated with breast cancer incidence. Similar findings were also obtained regarding calcium intake [37,41]. Specifically, Genkinger et al. [37], by analyzing 52,062 African American women [37], found no association between breast cancer and calcium intake (RR = 1.10, 95% CI = 0.79–1.53 comparing ≥1000 to <200 mg/day; *p* = 0.51). Similarly, Li et al. [41], in a cohort study involving 34,028 Singapore Chinese women, found no association between breast cancer and calcium intake irrespective of the source of consumption (i.e., from vegetables, dairy products, grains, or soy foods).

However, these investigations primarily employed food frequency questionnaires (FFQs) and interviews. It is crucial to acknowledge the methodological vulnerability of recall bias and measurement error. Further complexities are introduced with variable follow-up periods and inconsistent adherence to the investigated diets across participants. In light of these challenges, the initial premise linking dairy intake to breast cancer development is certainly not confident. Hence, further investigation and the formulation of precise guidelines are definitely needed.

**Table 2:** Main findings on the association between the consumption of dairy products and breast cancer incidence.

Author (Year) [Ref]	Food/Intervention under Investigation	Type of Study	Participants	Assessment of Dietary Intake	Main Results
Couto E et al. (2013) [33]	Mediterranean diet	Prospective cohort study	44,840 women	FFQ	A statistically significant inverse association was reported between dairy consumption and breast cancer risk in all pre-menopausal and post-menopausal women.
Genkinger JM et al. (2013) [37]	Dairy, Ca, Vit D, and meat consumption	Prospective cohort study	52,062 African American women	FFQ	The authors observed no significant association between breast cancer and dairy intake. A negative association was shown between milk consumption and hormone receptor-negative subtypes.
Bahadoran Z et al. (2013) [38]	Dairy products	Case-control study	275 Iranian women	FFQ	An inverse correlation between breast cancer and dairy intake was found, especially for low-fat and fermented dairy products.
Li J et al. (2013) [41]	Calcium	Prospective cohort study	34,028 women	FFQ	A lack of association between calcium intake and breast cancer risk was observed, independently of the source of consumption.
Zang J et al. (2015) [32]	Dairy products	Systematic review and meta-analysis	1,600,312 participants	FFQ, diet questionnaires, and 24-h recall data interview	High and moderate dairy intake reduced breast cancer risk compared to low consumption.
Farvid MS et al. (2018) [36]	Dairy products	Observational study	90,503 pre-menopausal women for early adulthood and 44,264 women for adolescent	FFQ	A positive correlation emerged between dairy intake and hormone receptor-negative breast cancer in contrast to the negative one observed for hormone receptor-positive breast cancer subtypes.
Shin WK et al. (2019) [31]	Milk	Prospective cohort study	78,320 participants	Interviewer-administered semi-quantitive FFQ	In the pre-menopausal setting, a negative association between high daily intake of milk (≥1 serving/day) and breast cancer risk was observed compared to women with low milk consumption (<1 serving/week).
Marcondes LH et al. (2019) [35]	Animal food (red meat, poultry, fish, dairy, and egg)	Prospective cohort study	3209 participants	FFQ and physical examination	No association was observed between breast cancer and dairy consumption in post-menopausal women.
Key TJ et al. (2019) [40]	Alcohol, fruit, dietary fiber, meat, fish, milk, cheese, yogurt, eggs, vegetables, dairy protein, fat, carbohydrates, and free sugars	Prospective cohort study	691,571 post-menopausal UK women without previous cancer history	FFQ	The authors found no association between the consumption of different kinds of dairy products and breast cancer risk.
Fraser GE et al. (2020) [34]	Dairy and soy	Prospective cohort study	52,795 North American women	FFQ and structured 24-h dietary recalls for calibration study subjects	Increased risk of developing breast cancer in the 90th and 10th percentile of consumption of dairy products in both pre- and post-menopausal women. Increased risk of the development of hormone receptor-positive subtypes.
He Y et al. (2021) [22]	Dairy products (fermented, non-fermented, low-fat, and high-fat dairy products)	Meta-analysis	1,019,232 participants	FFQ, diet questionnaires, and home visits or in-depth interviews	The statistically significant protection of fermented dairy products was observed only in post-menopausal women. A statistically significant protective effect of low-fat products was shown solely in pre-menopausal women.
Aguilera-Buenosvinos I et al. (2021) [39]	Dairy products	Prospective cohort study	10,930 women	FFQ	A moderate consumption of dairy products (2–4 servings per day) was associated with decreased breast cancer incidence in the post-menopausal setting. A low intake (1–2 servings per day) of low-fat dairy products consumption reduced breast cancer risk in the pre-menopausal setting.

FFQ: food frequency questionnaire.

## 5. Soy Intake

Recently, the potential link between soy intake and breast cancer risk has become a topic of heightened interest. This concern stems from soy’s protein content, which includes phytoestrogens with estrogen-like properties. Given the well-established association between estrogens and breast cancer development, exploring the connection between soy intake and breast cancer incidence has emerged as a pivotal focus of research.

Several clinical trials have sought to elucidate the extent to which soy consumption may influence breast cancer incidence (Table 3). In a case-control study by Tan et al. [42] involving 7663 Malaysian women, intriguing results were found. A frequency of soy milk consumption greater than once per week demonstrated an inverse association with breast cancer incidence (OR 0.25, 95% CI 0.18–0.33, *p* < 0.001), as did soy product consumption (OR 0.40, 95% CI 0.33–0.48, *p* < 0.001). Partially in contrast with these findings, a large prospective cohort study [43] encompassing more than 300,000 participants did not reveal a significant association, either with a moderate consumption of soy (i.e., 14.4 mg/day, HR 1.03, 95% CI 0.87–1.22, *p* = 0.537) or with a high intake (i.e., 19.1 mg/day, HR 0.98, 95% CI 0.80–1.20, *p* = 0.537). However, in a subsequent meta-analysis combining these results with eight other prospective cohort studies, a 3% reduced risk of breast cancer development with each 10 mg/day increase in isoflavone intake was shown (HR 0.97, 95% CI 0.95–0.99). Similarly, a meta-analysis by Boutas et al. [44], analyzing seven studies and involving a total of 485,495 participants, suggested an increased breast cancer risk with soy intake between 0 and 15 mg/day compared to a higher intake (OR 7.01, 95% CI 6.58–7.47, *p* < 0.001). In line with these results, a subsequent meta-analysis [45], including 10 studies and a case-control study involving 1120 participants [46], showed an inverse association between high soy consumption and breast cancer risk. Specifically, in the meta-analysis [45], the authors observed an inverse association between soy food consumption and breast cancer incidence (RR 0.92, 95% CI 0.84–1.00 and RR 0.91, 95% CI 0.84–1.00, for high versus low- and dose-response analyses, respectively). Similarly, Li et al. [46] showcased an inverse association between high total isoflavone intake and breast cancer development in both Chinese hospital outpatients and the general population (for isoflavone > 35.12 mg OR 0.52, 95% CI 0.33–0.85, *p* = 0.02 and OR 0.45, 95% CI 0.27–0.75, *p* < 0.01, respectively).

In addition to examining the quantity of soy consumed, numerous studies have been conducted with a specific emphasis on the origin of soy, yielding inconsistent findings. Shin’s meta-analysis [47], which included 15 cohort studies and 34 case-control studies, indicated no association between soy food intake and breast cancer development (RR 0.90, 95% CI 0.67–1.20), while the consumption of soy isoflavones was associated with a 32% reduction in breast cancer risk (RR 0.68, 95% CI 0.55–0.82), albeit with high heterogeneity in both analyses (I^2^ = 71.6% *p*= 0.001 and I^2^ = 62.8% *p* = 0.004, respectively). In contrast, a 35% decrease in breast cancer risk was observed with higher soy protein consumption (RR 0.65, 95% CI 0.51–0.83), displaying negligible heterogeneity (I^2^ = 45.0% *p* = 0.122). Similarly, a meta-analysis by Zhao and colleagues [48], including 16 prospective cohort studies and a total of 648,913 participants, showed no association between breast cancer risk and high or moderate isoflavones intake (for high versus low intake RR 0.99, 95% CI 0.91–1.09, *p* = 0.876 and for moderate versus low RR 0.99, 95% CI 0.92–1.05, *p* = 0.653). The authors also demonstrated an inverse association between high soy consumption and breast cancer risk compared with low consumption (RR 0.87, 95% CI 0.76–1.00, *p*= 0.048). On the other hand, they found that a moderate intake was not associated with breast cancer development (RR 0.93, 95% CI 0.82–1.07, *p* = 0.323) compared to a low intake. In contrast, Wang’s meta-analysis [49], encompassing two cohort and twelve case-control studies, revealed a protective effect of tofu, independently of menopausal status (for pre-menopausal women: ten studies included, OR 0.70, 95% CI 0.52–0.87, *p* < 0.001 and for post-menopausal: nine studies included, OR 0.72, 95% CI 0.47–0.97, *p* < 0.001). In addition, after a deeper analysis of six case-control studies, the authors underlined the dose-dependent protection of tofu, with each 10 g of tofu intake leading to a 10% reduction in breast cancer risk (OR 0.90, 95% CI 0.87–0.93, *p* = 0.037).

Within the investigations on the potential impact of soy on breast cancer risk, the role of menopausal status remains inconclusive. Specifically, conflicting results have been obtained when analyzing the effect of isoflavones on breast density, which is known to be a breast cancer risk factor in both pre-menopausal and recently menopausal women [24,50]. In fact, in the pre-menopausal setting, a randomized, double-blinded placebo-controlled clinical trial [24] involving 194 pre-menopausal women showcased a potential preventive effect of isoflavone intake, as the authors demonstrated a time-dependent effect of isoflavones in the isoflavone group compared to the placebo group, which led to an up to a 19.3 cc reduction in fibroglandular breast tissue (FGBT; 95%CI = −8–47) and up to a 3.5 cc decrease in breast tissue density (i.e., FGBT%: FGBT as percentage of breast tissue; 95% CI = −0.11–7.12). On the other hand, Rajaram et al. [50], in a clinical trial examining the effects of isoflavone intake through external supplementation and dietary sources, observed that a moderate (18–61 mg/d) and high (>61 mg/d) consumption of isoflavones reduced mammographic density up to 6 cm^2^, especially in recently menopausal women (i.e., women with a menopausal status less than six years) (−5.9 cm^2^ vs. −1.1 cm^2^ in the ISF diet arm and −0.8 cm^2^ in the control arm), a result, however, that was not statistically significant (*p* = 0.13). Besides these inconclusive findings, other studies have shown the protective effect of soy and isoflavones in correlation with menopausal status. In fact, in a prospective cohort study by Wada et al. [23] involving 15,607 women, the authors observed a protective effect of soy and isoflavone solely in post-menopausal women (HR 0.65, 0.67, and 0.63 in Q2, Q3, and Q4, respectively, *p* = 0.023 for soy intake and HR 0.57, 0.68, and 0.52 in Q2, Q3, and Q4, respectively, *p* = 0.046 for isoflavone). On the other hand, in two meta-analyses [51,52], this protective effect was independent of menopausal status. In particular, in a meta-analysis of 35 studies by Chen et al. [51], the authors demonstrated a statistically significant protective effect of isoflavone intake in both pre-menopausal and post-menopausal women but only those from Asian countries (OR 0.59, 95% CI 0.48–0.69 and OR 0.59, 95% CI 0.44–0.74, respectively), although a high degree of heterogeneity was found (I^2^ 53.2% and 809.7%, respectively). Similarly, a meta-analysis by Woo and colleagues [52] showed a protective effect of all the investigated soy products for Korean women, independently of their menopausal status, with the strongest inverse association for soybean curd (OR 0.47, 95% CI 0.34–0.66 for soybean curd, and OR = 0.75, 95% CI = 0.57–0.98 for soymilk). Nevertheless, it is crucial to consider that the present meta-analysis solely incorporated three studies.

Exploring the impact of soy on specific breast cancer molecular subtypes has revealed nuanced insights. Noteworthily, two recent studies suggested a protective effect of soy against the ER-negative breast cancer subtype, especially in pre-menopausal patients [53,54]. In detail, a meta-analysis by Okekunle et al. [53], including five cohort studies and thirteen case-control studies, showed a negative association between higher soy consumption and breast cancer risk, especially in pre-menopausal women (OR 0.79, 95% CI 0.71–0.87, *p* < 0.001) and with regards to the development of ER-negative subtypes (OR 0.71, 95% CI 0.57–0.90, *p* = 0.013). Consistent with the aforementioned results, Cao and colleagues [54] conducted a study on 1753 Chinese women to investigate the potential protective effects of a modified version of the Mediterranean diet known as the Chinese vegetable-fruit-soy diet. This diet substitutes olive oil, legumes, and whole grains with soy, rapeseed oil, and coarse cereals. They demonstrated a protective effect of this kind of diet in post-menopausal women (OR 0.57, 95% CI 0.41–0.80, *p* < 0.001) and, especially in this population, a protective effect against ER-negative (OR 0.63, 95% CI 0.37–0.94, *p* = 0.003) and ER- and PgR-negative breast cancer subtypes (OR 0.64, 95% CI 0.41–0.93, *p* = 0.012). The authors of both research studies speculated that the specific association with ER-negative breast cancer may be due to a preferential link to estrogen receptor beta compared to alpha. However, the authors did not exclude that the strong influence of hormonal factors in ER-positive tumors may have impaired the evaluation of dietary factors on cancer incidence.

A case-control study by Ko and colleagues [55] assessed whether specific food may prevent breast cancer development in women with known breast cancer (BRCA) gene pathogenic variants. The participants were selected from the Korean hereditary breast cancer study and included both high-risk subjects and known cancer patients bearing BRCA gene pathogenic variants. Through the use of a FFQ, they showed that following a soy-based diet reduced the chance of breast cancer development in BRCA gene pathogenic variant carriers (*n* = 419, HR 0.39, 95% CI 0.19–0.79 for the highest quartile, *p* = 0.005). This observation was more prominent for BRCA2, with high soybean consumption (4–5 servings/week) (*n* = 201, HR 0.38, 95% CI 0.16–0.93, *p* = 0.022). Differently, for BRCA1, breast cancer risk and soy-based diet were not associated. This result is partially in contrast with those reported above concerning the association between soy intake and ER-negative breast cancer since BRCA1 mutation carriers typically develop this specific tumor subtype. However, the observation by Ko and colleagues may be attributed to the small number of participants with the BRCA1 mutation.

In summary, the existing body of research paradoxically suggests an inverse correlation between soy intake and breast cancer development. However, demographic characteristics, different soy isoflavone components, and patient-related factors could significantly influence these results. Consequently, further research is imperative to delineate the precise association between soy, isoflavones, and breast cancer development.

**Table 3:** Main findings on the association between soy intake and breast cancer risk.

Author (Year) [Ref]	Food/Intervention under Investigation	Type of Study	Participants	Assessment of Dietary Intake	Main Results
Wada K et al. (2013) [23]	Soy and isoflavones	Prospective cohort study	15,607 women	FFQ	A negative association between soy and isoflavone intake and breast cancer risk was observed solely in post-menopausal women.
Li L et al. (2013) [46]	Isoflavone	Case-control study	1120 controls	FFQ	A protective effect of dietary isoflavone intake on breast cancer development was reported for both hospital outpatient and population controls.
Ko KP et al. (2013) [55]	Soy, vegetables, fruit, meat, and seafood	Case-control study	2271 women	FFQ	Negative association between soy consumption and breast cancer risk in BRCA carriers
Chen M et al. (2014) [51]	Soy and isoflavone	Meta-analysis	1,391,524 pre-menopausal and 579,33 post-menopausal women	n.d.	An inverse association was found between soy isoflavone intake and breast cancer incidence, independently of menopausal status, solely in Asian women.
Woo HD et al. (2014) [52]	Soy products, fruits, and vegetables	Meta-analysis	8112 participants	n.d.	Different kinds of soy foods were inversely associated with breast cancer risk in both pre-menopausal and post-menopausal women.
Wu J et al. (2016) [45]	Meat, soy, milk, yogurt, poultry, fish, eggs, and nuts	Meta-analysis	452,916 participants	n.d.	Reduced breast cancer risk with high soy consumption.
Zhao TT et al. (2017) [48]	Soy and isoflavone	Meta-analysis	648,913 participants	FFQs, self-administered questionnaires, and mail survey questionnaires	A statistically significant inverse association was shown between high versus low soy consumption and breast cancer risk.
Tan MM et al. (2018) [42]	Soy, breastfeeding, and PA	Case-control study	7663 women	Interviews and FFQs	High soy milk and soy product consumption demonstrated an inverse association with breast cancer incidence.
Wei Y et al. (2020) [43]	Soy and isoflavones	Prospective cohort study and meta-analysis	30,0852 women for the cohort study and 513,313 participants for the meta-analysis	FFQs, physical measurements, resurveys, 24-h dietary recalls	The cohort study revealed no association between moderate or high soy consumption and breast cancer. The meta-analysis showed a 3% reduced risk of breast cancer development with each 10 mg/day increase in isoflavone intake.
Wang Q et al. (2020) [49]	Tofu	Meta-analysis	109,813 participants	n.d.	A protective effect of tofu consumption on breast cancer development was observed independent of menopausal status.
Okekunle AP et al. (2020) [53]	Soy and isoflavone	Meta-analysis	29,810 participants	n.d.	Increased soy consumption reduced breast cancer risk, especially in pre-menopausal women and for ER-negative subtype development.
Lu LW et al. (2022) [24]	Isoflavones versus placebo	Clinical trial	194 pre-menopausal women	N.A.	The authors found a decrease in breast tissue density with higher isoflavone intake, especially in pre-menopausal women.
Boutas I et al. (2022) [44]	Soy and isoflavones	Meta-analysis	485,495 participants	FFQ	High soy consumption reduced the breast cancer risk in pre- and post-menopausal women.
Cao S et al. (2022) [54]	Vegetable-fruit-soy dietary pattern	Case-control study	1753 women	FFQ	Higher soy consumption reduced breast cancer development in post-menopausal women, especially ER- and ER-/PgR-negative subtypes.
Shin S et al. (2023) [47]	Fruits, vegetables, meat, soy, green tea, alcohol	Meta-analysis	216,216 participants	n.d.	A protective effect of soy protein and isoflavone intake on breast cancer incidence was observed, but no correlation was found with soy food consumption.
Rajaram N et al. (2023) [50]	Soy isoflavone supplement versus isoflavones from dietary sources	Clinical trial	90 women	FFQ	Moderate and high intake of soy reduced mammographic density in both pre-menopausal and recently menopausal women.

## 6. Discussion

Overall, our literature research highlights a protective effect of dairy and soy consumption in breast cancer development, whereas limited evidence was found on the association between high sugar intake and breast cancer incidence. The relationship between nutrition and the occurrence of breast cancer appears to be influenced by menopausal state and exhibits different correlations based on the molecular subtype. Elevated consumption of sugar was associated with a higher likelihood of developing breast cancer, particularly among post-menopausal women. This correlation is shown specifically in relation to diets that are high in GI and involve a significant intake of carbohydrates. Furthermore, research has shown a potential correlation between breast cancer subtypes that do not express hormone receptors and higher GI diets. Some cohort studies have documented a higher occurrence of breast cancers in those who consume sugar-sweetened drinks and artificial sweeteners. Nevertheless, studies conducted in this field have shown conflicting results, and there is a lack of agreement regarding the optimal level of sugar consumption.

In contrast, several cohort studies suggested that higher dairy intake, especially milk, may be protective. This association appeared more robust in pre-menopausal women. Additionally, fermented dairy products, such as yogurt and cheese, showed potential benefits in reducing breast cancer risk, but non-fermented dairy yielded inconsistent results, especially when considering intake quantity.

Lastly, soy intake has a multifaceted implication in breast cancer incidence due to its phytoestrogen content. Meta-analyses and prospective studies showed an inverse correlation between soy intake and breast cancer risk in post-menopausal women. Particular soy components, including soy isoflavones and soy protein, have demonstrated encouraging outcomes in the prevention of breast cancer incidence. However, this association may differ according to the molecular subtype, and conflicting findings have emerged; some studies suggested a positive association with hormone receptor-positive subtypes, while others leaned towards hormone receptor-negative subtypes.

Inconsistencies in these studies may result from single-study bias and methodological inaccuracies. In fact, in certain cohort studies, the authors mainly relied on questionnaires and interviews for data collection, thus increasing the risk of recall bias and measurement error. Participant follow-up and features like demographics, genetic factors, and specific components of sugar, dairy, and soy intake may further contribute to research complexity. Moreover, the complicated nature of examining and classifying dietary components posed challenges, given the absence of an internationally standardized categorization. Consequently, individual authors employed distinct classification systems, leading to variations in their measurement units and interpretations. Finally, individual metabolic responses to food may further influence the presented findings. For instance, high interpersonal variability in post-prandial glycemic responses was demonstrated, which may be due to patients’ biological and lifestyle factors [56]. As an example, the PREDICT 1 clinical trial [57] showed high individual differences in post-prandial responses to the same meal either among unrelated adults or between twins.

Finally, since we performed a narrative review aimed at qualitatively summarizing the currently available knowledge, a quantitative estimation of the effect of sugar, dairy, and soy consumption on breast cancer incidence through a meta-analysis was not feasible. Thus, future systematic reviews and meta-analyses are definitely needed to obtain uniform consensus for the incorporation of recommendations about dietary patterns into international guidelines.

## 7. Conclusions and Future Perspectives

Our narrative review highlights a negative association between breast cancer incidence and both dairy product and soy consumption, although patient- and cancer-related factors could significantly influence the protective effect of these foods. In fact, this review outlines the variability in outcomes based on menopausal status and differentiates between the types of dairy products. Regarding soy intake, conflicting findings related to soy’s impact on breast cancer risk are presented (limited data were available regarding the positive association between soy consumption and luminal-like breast cancer). Methodological vulnerabilities, such as recall bias and measurement errors, within the current body of research are highlighted, underscoring the imperativeness of further investigation into the intricate interplay between diet and breast cancer.

In contrast, limited evidence was found on the association between high sugar intake and breast cancer incidence. Therefore, the complete elimination of this nutrient appears excessive and possibly not beneficial. The multifaceted and evolving nature of the relationship between sugar intake and breast cancer is a relevant key factor. To navigate this complex landscape, a compelling call for well-designed prospective studies and trials emerges.

Our analysis reveals a nuanced scenario with potential protective effects of dairy and soy consumption against limited evidence regarding high sugar intake. The overarching theme of complexity underscores the necessity for continued research to unravel these relationships. Emphasizing this complexity, our call for further investigation aligns with the imperative to formulate precise guidelines for breast cancer patients and clinicians, acknowledging the intricacies involved in crafting targeted preventive strategies for breast cancer.

The next step involves defining a research program aimed at studying the impact of diet after cancer diagnoses (i.e., in breast cancer survivors), with a focus on secondary prevention and potential interactions with oncological treatments. A recent randomized controlled trial demonstrated that prescribing exercise and a healthy diet could be associated with a higher pathological complete response rate in breast cancer patients undergoing neoadjuvant treatment [58]. Similarly, recent observations suggest that a less pro-inflammatory baseline diet may be associated with better intestinal health and, in turn, an improved response to pre-operative therapy [59]. Moreover, new prospective studies are evaluating the impact of caloric restriction in the neoadjuvant setting [60] or the fasting-mimicking diet in the advanced setting [61]. These findings suggest that dietary interventions have the potential to play a significant role in improving outcomes for patients with breast cancer. Further research is needed to better understand the specific mechanisms by which diet influences cancer development and progression, as well as to identify the optimal dietary strategies for prevention and treatment. By gaining a deeper understanding of the complex interplay between diet and cancer, we can develop more personalized approaches to enhance breast cancer prevention in healthy individuals and support patients in their fight against breast cancer.
